# Decay stages of Jurassic wood debris from Scotland: evidence for the coevolution of fungal rot, arthropods and the nurse log strategy

**DOI:** 10.1111/nph.70515

**Published:** 2025-09-08

**Authors:** Ana Julia Sagasti, Sophie Burne, Jeremy Wyman, Alexander J. Hetherington

**Affiliations:** ^1^ Institute of Molecular Plant Sciences, School of Biological Sciences University of Edinburgh Max Born Crescent Edinburgh EH9 3BF UK; ^2^ CONICET and Facultad de Ciencias Naturales y Museo, Universidad Nacional de La Plata Instituto de Recursos Minerales (UNLP‐CICBA) La Plata B1904AMA Argentina; ^3^ Royal Botanic Garden Edinburgh Edinburgh EH3 5LR UK; ^4^ Department of Natural Sciences National Museums Scotland Edinburgh EH1 1JF UK

**Keywords:** conifers, fossil fungi, fungal decay, palaeobotany, plant evolution, plant–arthropod interactions, plant–plant interactions, roots

## Abstract

- A key feature of extant conifer forests is the high percentage of seeds that germinate and establish on dead wood; in some forests, this can exceed 90%. This deadwood can act as an ideal nursery for young tree species, leading to this type of seedbed being termed ‘nurse logs’. It is unclear how common this ecological strategy has been throughout the evolutionary history of conifers.
- Here, we describe a *c*. 150‐million‐year‐old wood fragment from the Jurassic of Scotland using classic palaeobotanical techniques and microscopy. We interpret our new finding within the evolution of gymnosperms with a literature review of fossil nurse logs, and fungal and arthropod evolution.
- We interpret the timeline of decay processes on this log as consisting of three main stages: breakdown of the wood by white pocket rot; two types of arthropod boring, likely by beetles and mites; and colonisation by small roots. These findings demonstrate the complexity of decay processes occurring on a Jurassic forest floor, and the key role that white rot and arthropods play in the nurse log ecological strategy.
- Conifer germination on nurse logs facilitated by white rot fungi is a conserved ecological strategy of relevance in shaping forest ecosystems over geological timescales.

## Resumen

- Los bosques modernos de coníferas se caracterizan por el alto porcentaje de semillas que se establecen y germinan en fragmentos de madera. En algunos bosques, este porcentaje puede exceder el 90%. Los fragmentos de madera constituyen un substrato ideal para el establecimiento de árboles jóvenes en lo que se ha denominado ‘*nurse logs*’. Sin embargo, no está claro qué tan común ha sido esta estrategia ecológica a lo largo de la historia evolutiva de las coníferas.
- En este trabajo describimos un fragmento de madera proveniente del Jurásico de Escocia (*ca*. 150 millones de años) utilizando técnicas clásicas de paleobotánica y microscopía. Los resultados de este análisis son interpretados en el contexto de la evolución de las gimnospermas a través de una revisión bibliográfica enfocada en el registro fósil de los ‘*nurse logs*’ y la evolución de hongos y artrópodos.
- Realizamos una reconstrucción del proceso de decaimiento del fragmento de madera que consiste de tres etapas principales: desgaste de la madera por acción de pudrición blanca, excavación de galerías de artrópodos, probablemente escarabajos y ácaros, y por último el establecimiento de raíces pequeñas. Nuestro análisis demuestra la complejidad de los procesos de decaimiento que ocurrieron en el suelo de un bosque jurásico, y el rol clave que representan la pudrición blanca y la actividad de artrópodos en la estrategia ecológica ‘*nurse log*’.
- Nuestros resultados sugieren que la germinación de semillas de coníferas en ‘*nurse logs*’ se ve facilitada por la actividad de podredumbre blanca y representa una estrategia ecológica de relevancia en la conformación de ecosistemas forestales a lo largo del tiempo geológico.

## Introduction

Conifers are the most diverse and widespread group of extant gymnosperms and have a fossil record extending over 300 million years (Myr). They have been key components of terrestrial ecosystems since at least the late Carboniferous (Plotnick *et al*., 2009; Leslie *et al*., 2018). Conifers have a global distribution and, despite the rise of angiosperms, are still major components of temperate and boreal forests today (Farjon & Filer, 2013; Farjon, 2017). Their long evolutionary history and global diversity contribute to an excellent fossil record, providing insights into the evolution of forest ecosystems through time. They also represent the main component of many of the world's famous fossil forests known from the geological record (e.g. Wieland, 1935; McKnight *et al*., 1990; Mosbrugger *et al*., 1994; Del Fueyo *et al*., 1995; Fairon‐Demaret *et al*., 2003; Falaschi *et al*., 2011; Correa *et al*., 2019; Gee *et al*., 2019, 2023; Gou *et al*., 2023; Trümper *et al*., 2023). Although fossils provide substantial evidence for the size and distribution of trees and extensive records of fossil wood, they often provide limited evidence for the complex ecological processes that characterise modern forests. For example, the fossil record of the crucially important process of regeneration, where germination and growth of new trees provide long‐term continuity to forests, is scant.

### Seedling establishment and regeneration in modern forests

Seedling establishment is a challenging process in modern forests, including those dominated by conifers. The thick litter layer that forms the upper horizon of soils can become a barrier for the establishment of tree species, especially those with small seeds (Fukasawa, 2012, 2016). Additionally, exposure to soil pathogens, shading by floor vegetation and root competition can prevent the growth of seedlings (Coomes & Grubb, 2000; Fukasawa, 2016). Plants have therefore resorted to alternative seedbeds to improve the chances of establishment. Dead wood – either in the form of fallen trees or branches – can provide a valuable substrate for seeds to germinate and take root. The role of dead wood is crucially important in modern forests, leading to the origin of the term ‘nurse log’ to describe fallen wood that acts as a nursery for seedlings (Franklin & Dyrness, 1973; Harmon *et al*., 1986; Lack, 1991). Nurse logs are key features of boreal (Hofgaard, 1993), temperate (Harmon & Franklin, 1989; Christie & Armesto, 2003) and tropical forests (Lack, 1991; Sanchez *et al*., 2009), and are particularly important in conifer forests (Bače *et al*., 2012; Fukasawa, 2012, 2016; Woods *et al*., 2021). The enhanced survival of seedlings, especially of conifer species, on decaying deadwood has been widely reported in forests around the world (e.g. McKee *et al*., 1982; Harmon *et al*., 1986; Harmon & Franklin, 1989; Lonsdale *et al*., 2008). Quantification of *Picea* and *Tsuga* seedlings in a mature forest in Olympic National Park, USA, by McKee *et al*. (1982) demonstrated that over 95% of juvenile plants were established on nurse logs. By contrast, < 3% of young plants were on ground humus (McKee *et al*., 1982). Studies like this demonstrate the importance of nurse logs in promoting seed germination by a combination of factors such as moisture retention, mineral recycling, isolation from soil pathogens and provision of mycorrhizal fungi (Lonsdale *et al*., 2008). Due to the impact that nurse logs have on plant survival, especially their function in conifer forests, this has motivated palaeobotanists to investigate the nurse log fossil record to characterise the evolution of this strategy through time.

### The fossil record of nurse logs

Nurse logs are challenging to identify in the fossil record, as few depositional settings enable the preservation of germinating seedlings on deadwood from the forest floor. Palaeobotanists have instead concentrated on identifying decaying fossilised wood containing roots, providing direct evidence that deadwood is acting as a substrate for other plants (e.g. Daugherty, 1963; Césari *et al*., 2010, 2012; Decombeix *et al*., 2020; Feng *et al*., 2022; Vera & Perez Loinaze, 2022; Wang *et al*., 2024; Molano *et al*., 2025). Characterising fossil wood containing roots as a nurse log will not always map entirely to the concept of modern nurse logs, as it can be challenging to establish whether the roots were those of seedlings or more mature plants, or whether woody fragments were partially or fully buried in the substrate before being colonised by roots. However, in both cases they provide valuable evidence of wood acting as a substrate for the growth of other plants. The ability to identify these plant–plant interactions requires an exceptional level of preservation of small roots within larger wood fragments. This limits the study of fossil nurse logs to two broad settings: (1) fossil peats where decaying wood is preserved *in situ* in the substrate, or (2) isolated fragments of permineralised wood. Combining evidence from these two sources demonstrates that nurse log type interactions are ancient and can be identified throughout the geological record, including the Carboniferous (Césari *et al*., 2010, 2012; Wang *et al*., 2024), Permian (Decombeix *et al*., 2020; Feng *et al*., 2022), Triassic (Daugherty, 1963), Jurassic (Molano *et al*., 2025), Cretaceous (Vera & Perez Loinaze, 2022) and Eocene (Fairon‐Demaret *et al*., 2003). However, despite this broad geological range spanning a 300‐Myr time interval, fossil nurse logs have been described from only eight fossil localities. Due to the important role that nurse logs play in modern conifer forests, extending their fossil record and identifying the genesis of these nurseries is key to understanding their evolutionary history and their impact on forest ecology through time.

Studying nurse logs is also valuable as they provide vital examples of the processes of wood decay in the past. Dead wood contributes to the structural diversity of forest ecosystems and has a major role in nutrient cycling and carbon dynamics (Kruys *et al*., 2002). Most of the carbon present in dead wood is returned to the ecosystem by multi‐trophic decay and breakdown processes carried out by fungi, bacteria and microarthropods. However, there is still limited direct evidence for the evolution of wood decay processes through the geological record (e.g. Creber & Ash, 1990; Tanner & Lucas, 2013; Gnaedinger *et al*., 2015; Sagasti *et al*., 2019; Sagasti & Bodnar, 2023; Cai *et al*., 2024) in comparison with the number of studies of fossil woods themselves (e.g. Pool & Cantrill, 2006; Taylor *et al*., 2009; Trümper *et al*., 2018; Philippe, 2023 and citations therein). Fossil nurse logs, therefore, additionally contribute valuable evidence for the complexity of food webs in the past and the interaction between plants, animals and fungi.

Here, we extend the fossil evidence for nurse logs with a description of, to our knowledge, the first example of a Jurassic (*c*. 150 million‐year‐old) nurse log from the northern hemisphere and only the second Jurassic locality globally. We report evidence for interactions with fungi, arthropods and plant roots within the wood fragment. This evidence supports the hypothesis that the sample was not buried before fossilisation and therefore suggests an ecology most similar to modern nurse logs. Finally, this nurse log provides evidence of rich ecological interactions in a conifer‐dominated forest of the Upper Jurassic of Scotland. Our new findings add to a growing body of evidence that the nurse log strategy is a common feature of conifer forests facilitated by the coevolution of wood‐rotting fungi and arthropods.

## Materials and Methods

### Geological setting

**Fig. 1:**
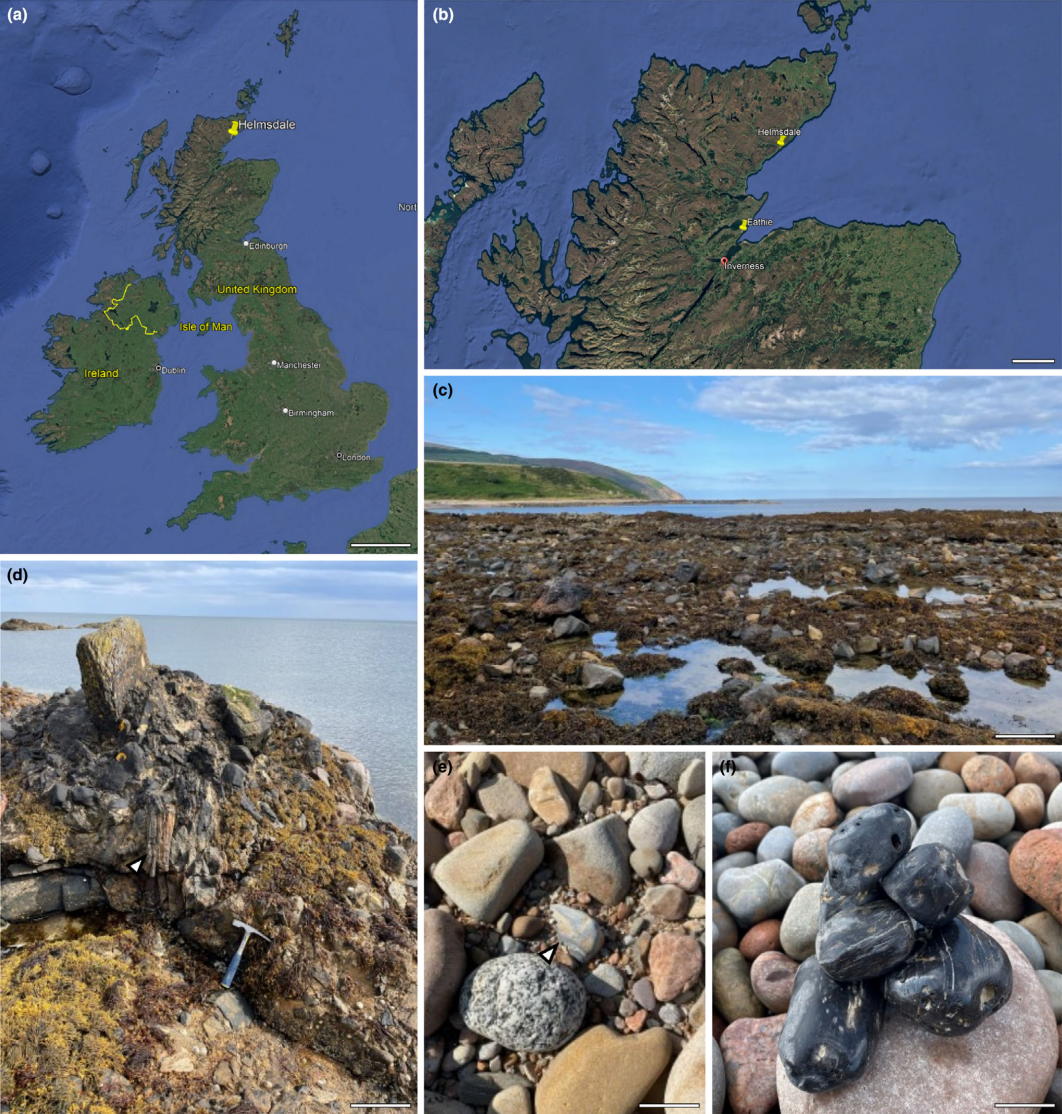
Geological setting of the Helmsdale Boulder Bed. (a) Map of the United Kingdom and Ireland showing the position of Helmsdale. (b) Detail of the north of Scotland showing the position of the main Jurassic localities that preserve permineralised woods. (c–f) Field photograph of the Helmsdale Boulder Bed (c) with examples of *in situ* fossil wood (d) and fossil wood eroded out and identified loose on the beach (e, f). Fossil wood was typically dark grey or black when wet. Bars: (a) 165 km; (b) 25 km; (c) 60 cm; (d) 30 cm; (e) 5 cm; (f) 3 cm.

Upper Jurassic rocks containing plant fossils outcrop on both the east and west coasts of Scotland (Riley, 1980; van Konijnenburg‐van Cittert & van der Burgh, 1996; Cleal *et al*., 2001; Nunn & Price, 2010; Cope, 2015; Smith & Strachan, 2024). These sites consist of deltaic and lagoonal environments with either transported or *in situ* plants. The largest number of sites is preserved along the east coast of Sutherland, where an 800‐m thick series preserves a Kimmeridgian–Tithonian (*c*. 155–145 million years ago (Ma)) succession with the oldest sediments at Kintradwell in the south and the youngest Dùn Glas in the north (Fig. 1a).

Of the east coast Jurassic sites, two stand out as possible locations to identify permineralised fossil nurse logs: Eathie and Helmsdale (Fig. 1b). Fossil plants at Eathie (Miller, 1857; Seward & Bancroft, 1913; Seward, 1911, 1919; Rothwell *et al*., 2011) are preserved in calcareous nodules, while the Helmsdale fossils preserve permineralised wood (Cleal *et al*., 2001). The abundance of permineralised fossils from the Helmsdale Boulder Beds, a rock series interbedded with marine shales that likely accumulated as a submarine fan (Pickering, 1984; Cleal *et al*., 2001), made it our priority search site.

Large pieces of fossil conifer wood are commonly found on the foreshore of Helmsdale, having weathered out of the boulder bed. These calcareous fossils have been described for over 160 yr (Miller, 1857). The first detailed description of the wood came in the early 20^th^ century (Seward & Bancroft, 1913; Seward, 1911, 1919). Seward & Bancroft (1913) described wood composed almost exclusively of tracheids with clear growth rings, with similarities to living *Araucaria*, and assigned it as a new species of *Cedroxylon hornei* (Seward & Bancroft, 1913). A further study of wood from the foreshore of Helmsdale was carried out by Creber in 1972. The study reported large fragments of wood, some over 30 cm in size, and determined the wood to be most similar to *Cedroxylon*, *Cupressinoxylon* and *Mesembrioxylon*. Despite the large amount of fossilised wood from Helmsdale, there have been no reports of nurse logs or descriptions of interactions between animals, plants and fungi often present in decaying wood. We therefore carried out new fieldwork to search for ecological interactions in the fossil wood.

### Sample collection and sample preparation

Loose fossil wood was collected along the foreshore northeast of Helmsdale (58°07′15.8″N, 3°37′48.6″W) in August 2023 and 2024 (Fig. 1c–f). Specimens were visually inspected under a stereoscope to select the most promising specimens for further analysis. Specimens were cut with a rock saw, and standard thin sections were prepared from transverse, radial longitudinal and tangential longitudinal sections. Thin sections and the remaining fragments from the hand samples were accessioned into National Museums Scotland.

### Photography and photogrammetry

Images of thin sections were taken using a Keyence VHX‐7000N microscope (Keyence Ltd, Milton Keynes, UK). The HDR Function of the Keyence VHX‐7000N was used to maximise image quality. Macro‐scale features of the nurse log were taken under cross‐polarised light with a Canon EOS 5D Mark IV camera (Canon Inc., Tokyo, Japan) and EF 100 mm f/2.8L Macro IS USM lens. This camera and lens set‐up was also used to capture the 3D structure of the nurse log using photogrammetry (Luhmann *et al*., 2013; James *et al*., 2023; Steffi, 2024). Photographs were taken of the specimen placed on a Genie mini II turntable within a 16″ × 16″ Neewer Studio Box lightbox. A scaled model and renders were created using AgiSoft Metashape Professional (64‐bit). The 3D model and associated images were uploaded onto the Edinburgh DataShare doi: https://datashare.ed.ac.uk/handle/10283/8998.

### Fossil description

Wood samples are described using the standardised format proposed by Boura *et al*. (2021) for fossil tracheidoxyls. Anatomical terminology follows IAWA Committee (2004) and Boura *et al*. (2021). A minimum of 30 measurements of each anatomical character was made using the Keyence VHX‐7000N software. Measurements are presented in Table 1 with minimum, maximum and average sizes. The classification criterion follows Philippe & Bamford (2008).

**Table 1:** Quantification of anatomical features of the fossilised conifer wood.

	Minimum	Maximum	Average
Earlywood tracheids			
Radial diameter (μm)	17	32	24
Tangential diameter (μm)	18	32	25
Double‐wall thickness (μm)	4	7	5
Latewood tracheids			
Radial diameter (μm)	6	15	9
Tangential diameter (μm)	9	27	16
Double‐wall thickness (μm)	4	8	6
Radial pits in tracheids			
Width (μm)	11	24	16
Height (μm)	9	25	14
Cross‐field oculipores			
Minor diameter (μm)	9	18	12
Major diameter (μm)	11	22	16
Ray height (no. of cells)	1	10	4
No. of rows of tracheids between rays in transverse section	2	17	6

## Results and Discussion

**Fig. 2:**
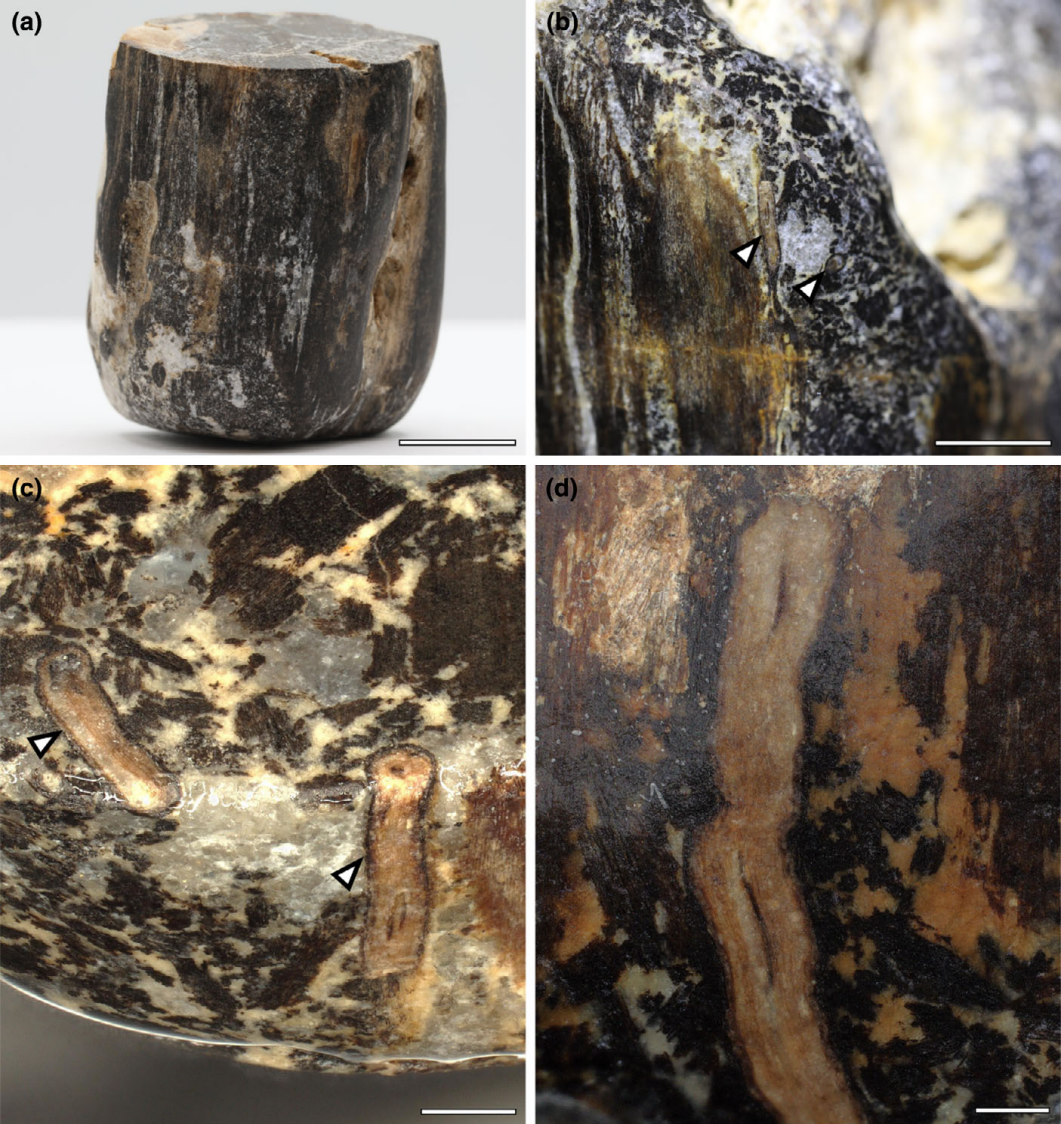
Surface view of the nurse log showing mottled coloured surface and fossil roots. (a–c) The mottled surface is due to the presence of frass and specimens preserve numerous roots (arrowheads). Most roots are oriented parallel to the main wood axis. (d) Detail of the root showing the preservation of cortex and vascular strand. Bars: (a) 1 cm; (b) 2000 μm; (c) 1000 μm; (d) 500 μm. Specimen accession codes: (a, b) Helmsdale 1 before sectioning; (c) NMS G.2025.5.2; (d) NMS G.2025.5.1.

Of the *c*. 20 specimens of fossil wood identified on the foreshore, one, we term Helmsdale 1 (NMS G.2025.5.1–5.9), was of particular interest. The specimen was 3 cm in diameter (Fig. 2a) and, unlike most specimens that were entirely black (Fig. 1f), this specimen had a mottled surface indicative of decay before premineralisation. Microscopic examination of the specimen demonstrated that within the woody axis, there was clear evidence of arthropod borings filled with small, irregular fragments of plant tissue (i.e. frass) (Fig. 2b–d), as well as small roots extending parallel to the main woody axis (Fig. 2b–d). Together, the presence of these features demonstrates evidence of nurse log‐like interactions in this piece of wood. Therefore, this warranted a full description of the main woody axis and the evidence for plant–plant, arthropod and fungal interactions.

### The nurse log was a conifer, likely *Cupressinoxylon* Göppert

**Fig. 3:**
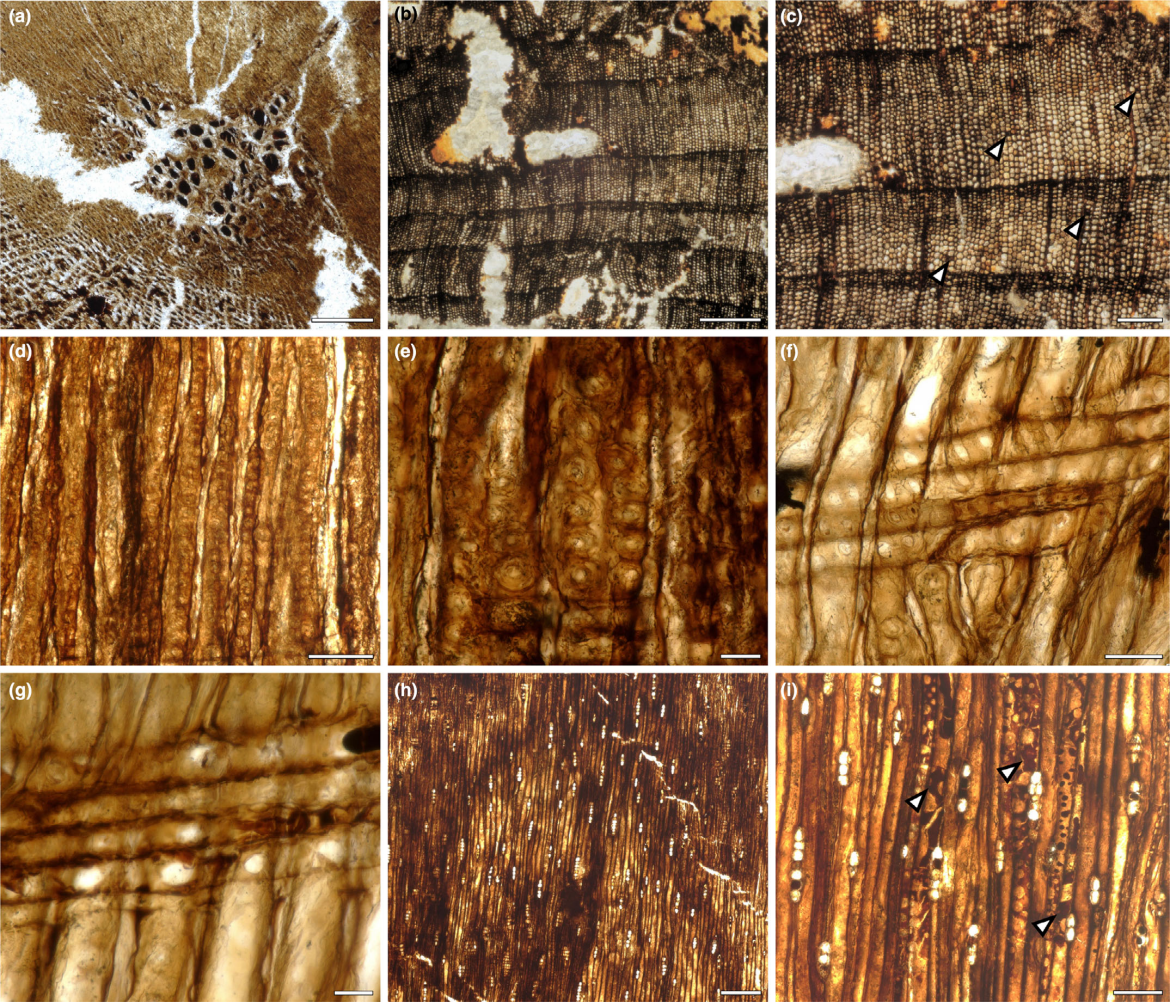
Anatomical features indicate that the wood was a conifer, likely *Cupressinoxylon* Göppert. (a) Homogeneous pith with dark‐filled parenchyma cells. (b) Transverse section of the log showing the presence of annual rings. (c) Detail of the annual rings and presence of axial parenchyma (arrowheads). (d) Radial longitudinal section showing the pits in tracheid walls. (e) Detailed view of the pits arranged contiguous to slightly compressed in one series, and with a bordered rim surrounding the circular aperture. (f) Cross‐fields with one to two oculipores of cupressoid type. (g) Steinkerne preservation of the fossil wood results in the loss of the pit's border and an apparent simple aperture. (h) General view of the tangential longitudinal section showing the presence of uniseriate rays. (i) Detail of the rays and axial parenchyma with dark contents (arrowheads). Bars: (a) 200 μm; (b) 500 μm; (c) 200 μm; (d) 100 μm; (e) 25 μm; (f) 50 μm; (g) 25 μm; (h) 250 μm; (i) 100 μm. Specimen accession codes: (a) NMS G.2025.5.8; (b, c) NMS G.2025.5.7; (d, e) NMS G.2025.7.5; (f, g) NMS G.2025.8.1; (h, i) NMS G.2025.6.5.

The nurse log preserves a woody axis with pith and secondary xylem tissues and represents either a branch or the internal central core of a trunk from a conifer (Fig. 3). The surface of the fossil specimen was worn and eroded, so it was not possible to estimate what size this fragment would have been in life. We prepared transverse and longitudinal sections to describe the anatomy of the wood to provide a tentative assignment of its affinity. Fossil woods, much like modern woods, are classified based on the types, size and shape of individual cells as well as tissue organisation (Philippe & Bamford, 2008; Boura *et al*., 2021). Gymnosperm wood is mainly composed of tracheids, with parenchyma as a secondary, less abundant component, and thus is termed homoxylous. Within the gymnosperms, two main types of woods are known: manoxylic, having wide rays with multiple rows of cells (e.g. cycads); and pycnoxylic, with narrow rays of one to three rows of cells (e.g. conifers, ginkgoes). The fossil studied here has a pith composed of parenchyma cells only, and these are filled with dark contents (Fig. 3a). Tracheids are the main cellular component of the secondary xylem (Fig. 3b,c). Axial parenchyma is scarce and appears scattered (Fig. 3c, arrowheads), and the rays are uniseriate (Fig. 3b,c); therefore, the wood is homoxylic and pycnoxylic. Growth rings are distinct, with an abrupt reduction of the radial diameter of tracheids in the transition from early‐ to latewood (Fig. 3b,c). Tracheids have bordered abietinean pits in their radial walls (Fig. 3d). These pits are round and arranged in one series; they are contiguous to slightly compressed and have circular apertures (Fig. 3e). The number, shape and disposition of pits (= oculipores) within the crossfield are of high taxonomic value in the classification of gymnosperm woods (IAWA Committee, 2004; Philippe & Bamford, 2008; Boura *et al*., 2021). In the fossils studied here, one to two oculipores with an oval outline, a bordered rim and an oblique aperture are present (Fig. 3f). In some areas, the oculipores appear to be big simple apertures without a border (Fig. 3g). This is a common result of the fossilisation process, called Steinkerne preservation, which results in the loss of the pit's border (Boura *et al*., 2021). Rays are composed of rectangular parenchyma cells only, defining them as homogeneous and homocellular (Fig. 3f,g). Rays are uniseriate and very low to medium in height following the IAWA definition, where very low constitutes up to four cells, and medium is between 5 and 15 cells (IAWA Committee, 2004), and do not have intercellular spaces between ray cells (Fig. 3h,i). The axial parenchyma is not abundant; its position within the secondary xylem is diffuse or associated with the rays and can present resiniferous contents (Fig. 3c,i, arrowheads). Based on this description of the wood and quantification in Table 1, we conclude that the woody axis of the nurse log was a conifer, most likely *Cupressinoxylon* Göppert.

The taxonomy of fossil woods has been used in descriptive terms to reference affinities with modern taxa (Philippe & Bamford, 2008; Boura *et al*., 2021). The fossil genus *Cupressinoxylon* has been used for conifer wood with anatomy similar to the recent *Cupressus* or Cupressaceae (Vaudois & Privé, 1971). However, some of the anatomical characters are also shared with the Podocarpaceae (Pujana & Ruíz, 2017; Ríos Santos *et al*., 2020). The lack of additional megafloristic elements (i.e. leaves, reproductive structures) for this area of Scotland prevents making speculations about the family affinities of the fossil woods from Helmsdale. The finding of this conifer wood is consistent with the presence of other conifer fossil woods described previously from Helmsdale. However, unlike other previously described fossils, this wood fragment contains abundant evidence of interactions with fungi, arthropods and plants after death.

### The first major decay process was the breakdown of wood by fungal activity consistent with white pocket rot

**Fig. 4:**
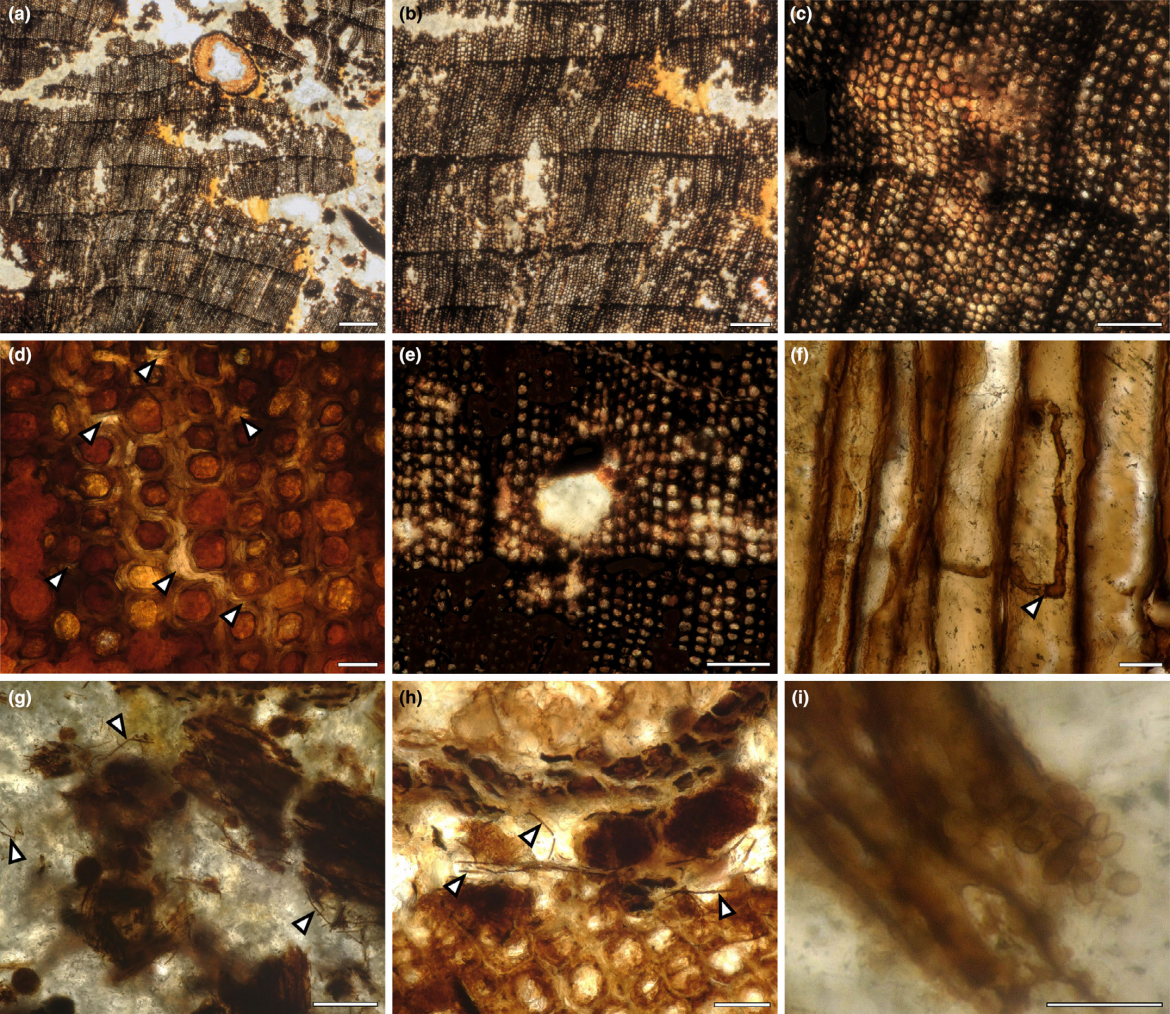
Wood preserves evidence of decay consistent with white pocket rot and fungal remains. (a, b) General decay of the wood showing the presence of pockets devoid of cells; (b) is a magnified image of (a). (c) Early stages of decay are evidenced by the whitening and thinning of cell walls. (d) Detached cells in the secondary tissue demonstrate the decay of the middle lamella (arrowheads). (e) More advanced decay leads to the development of rot pockets. (f) Fungal hyphae inside the decayed tracheids. The arrowhead points to transverse septa. (g) Thin hyphae (arrowheads) at the interior of the big gallery, surrounded by frass and coprolites. (h) Thin hyphae around roots and two coprolites (arrowheads). (i) Smooth‐walled, elliptic spores without ornamentation. Bars: (a) 500 μm; (b) 250 μm; (c) 100 μm; (d) 25 μm; (e) 100 μm; (f) 25 μm; (g) 100 μm; (h) 50 μm; (i) 20 μm. Specimen accession codes: (a) NMS G.2025.5.6; (b–e) NMS G.2025.5.7; (f) NMS G.2025.6.5; (g–i) NMS G.2025.5.7.

Specimen Helmsdale 1 preserves convincing evidence of a series of events from the death of the branch or trunk, followed by fungal decay, boring by arthropods and then invasion by roots. We hypothesised that the first stage was fungal rot, which would have broken down the wood and facilitated later stages of decay. Fungal rots are classified as brown‐, soft‐ and white‐rot according to the cell wall component that is primarily decayed by fungi (Blanchette, 1991; Schwarze *et al*., 2000; Schmidt, 2006; Schwarze, 2007; Krah *et al*., 2018; Goodell & Nielsen, 2023). Patterns of fungal rot observed in this study are characterised by decolouration and breaking of cell walls, indicative of the decay of all cell components (i.e. lignin, cellulose and hemicellulose) (Fig. 4a–e). The decay of the middle lamella, composed of pectin and lignin, can be identified by the detachment of adjacent cells (Fig. 4d, arrowheads). Progressive decay of cell walls, from S1 to S3, is evident in the secondary xylem (Fig. 4c,d). The less decayed tracheids show whitening of their cell walls, and more affected areas show patches of space where cells used to be (Fig. 4b,e). This patchy pattern is called white pocket rot (Schwarze *et al*., 2000; Schwarze, 2007; Harper *et al*., 2017; Goodell & Nielsen, 2023).

We searched for evidence of the fungi that caused this white pocket rot. In modern systems, white rot is mainly caused by the activity of Basidiomycete fungi and, to a lesser extent, by some Ascomycete fungi (Schwarze, 2007). We identified hyphae preserved in thin sections made from specimen Helmsdale 4 (NMS G.2025.6.1–6.5). Hyphae are simple, narrow (6–9 μm wide), with or without dark septa (Fig. 4f). These features are insufficient to determine their affinity, which in fossils would typically rely on the presence of asexual or sexual spores, complex spore‐producing organs and clamp connections (e.g. Taylor *et al*., 2015). Although we were not able to taxonomically classify these fungi, their presence in wood from Helmsdale, inside cells affected by white rot, suggests they could have caused the wood rot.

A second type of thin hyphae (2–4 μm wide) without septa abounds inside the bored nurse log and close to coprolites (Fig. 4g) and roots (Fig. 4h). These hyphae are darkly pigmented and lack conspicuous features of systematic value. Only a few smooth‐walled, elliptical spores, 4 (3.3–4.4) × 5.5 (4.6–5.7) μm in size were observed (Fig. 4i); these are not directly connected with hyphae and lack ornamentation, thus their affinity is uncertain. These hyphae are located within the arthropod boring and close to roots rather than inside the tracheid lumen (Fig. 4g,h). The second type of fungus might have been introduced by the arthropod or constitute an additional saprotrophic component.

### The wood was then bored extensively by arthropods

The most prominent feature of the external face of specimen Helmsdale 1 is a mottled appearance indicative of a large area having been excavated and bored by arthropods and now filled with frass (Fig. 2). Based on a combination of macroscopic and thin section observations, we described the evidence for arthropod damage to the woody axis. In the absence of body remains of arthropods, the identity of borers can be established based on the shape, size and contents of their galleries, the plant tissues affected by their activity and the shape, size, surface texture and content of the coprolites (e.g. Labandeira *et al*., 1997; Grimaldi & Engel, 2005; Toriti *et al*., 2021; Greppi *et al*., 2023). Using these features, we recognised distinct large and small galleries bored in the secondary xylem.

**Fig. 5:**
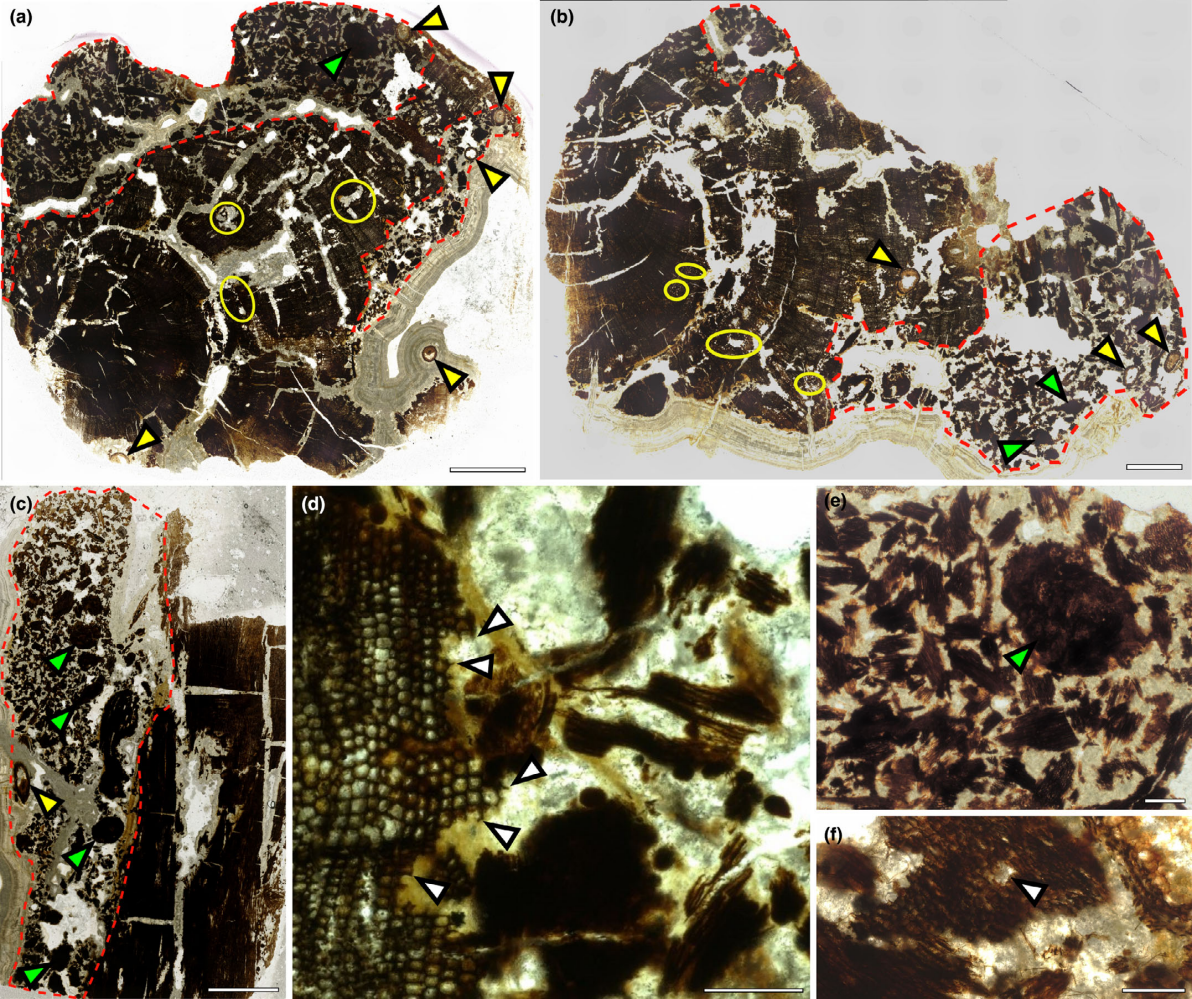
Transverse and longitudinal sections of the nurse log showing two types of arthropod borings and the presence of roots. (a, b) Decayed log with two big galleries filled with frass fragments (red dotted line), smaller galleries with irregular shapes filled with coprolites but not frass (yellow circles). Transverse section of roots (yellow arrowheads) and compacted frass (green arrowheads). (c) Longitudinal section of the log showing one big gallery (red dotted line) filled with fragments of frass, small coprolites and compacted ‘plugs’ of frass (green arrowheads). (d) Detail of the margin of the big gallery showing notched border (arrowheads). (e) Abundant frass inside the big gallery, including an area of compacted frass forming a ‘plug’ (green arrowhead). (f) Detail of frass fragment showing evidence of white pocket decay (arrowhead). Bars: (a–c) 2000 μm; (d) 200 μm; (e) 500 μm; (f) 200 μm. Specimen accession codes: (a) NMS G.2025.5.7; (b) NMS G.2025.5.6; (c) NMS G.2025.5.9; (d–f) NMS G.2025.5.7.

**Fig. 6:**
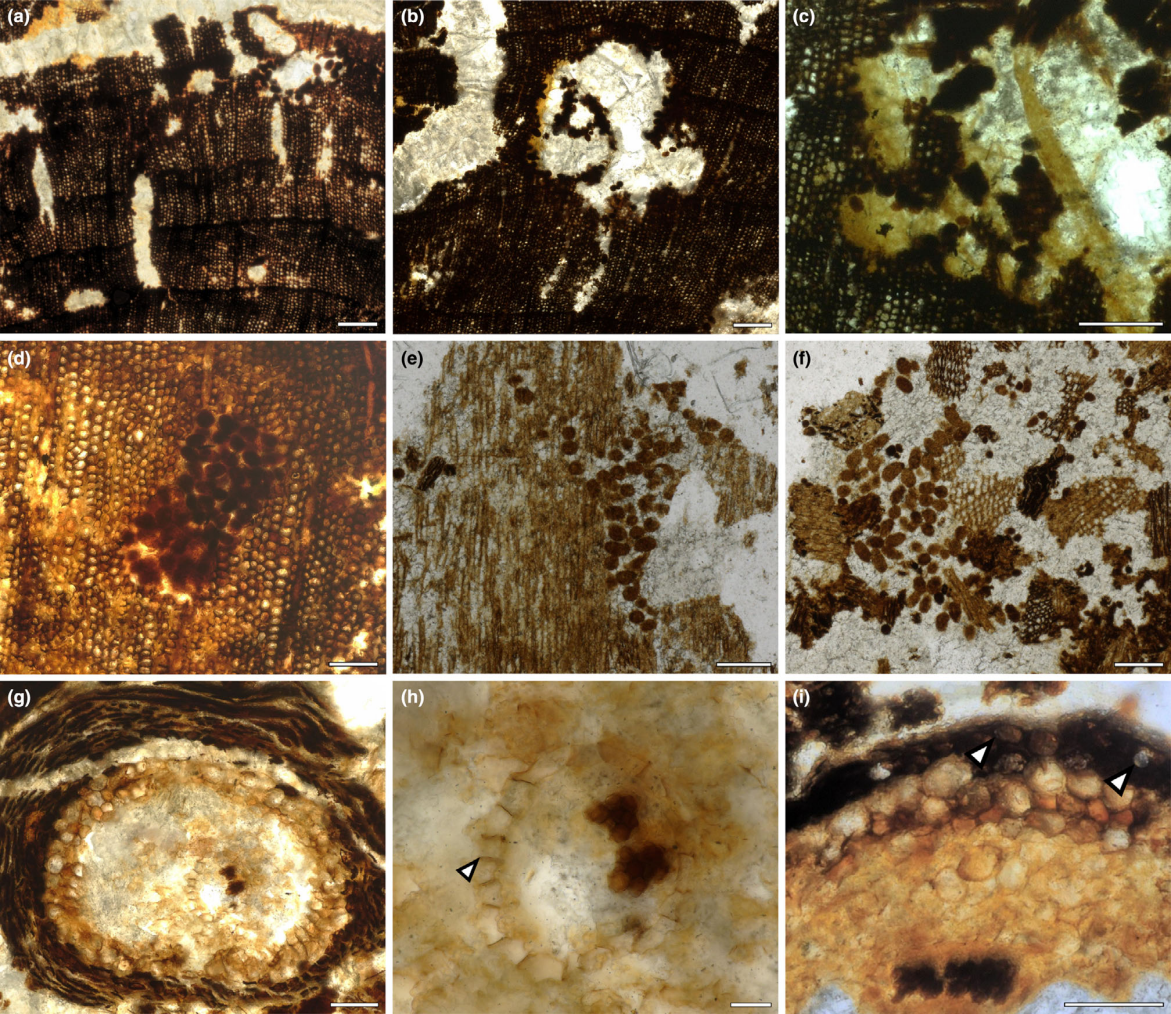
Details of arthropod damage, coprolites and root anatomy. (a) General view of the small, irregular galleries filled with coprolites but not frass. (b) Detail of one small gallery with loose coprolites and smooth margins. (c) Small galleries broken and coalescing with the main gallery. (d) Detail of a small gallery with packed class‐size I coprolites. (e) Longitudinal section of a small gallery with class‐size II coprolites. (f) Class‐size II elliptic coprolites mixed with frass fragments inside the main gallery. (g) Diarch root with vascular strand, outer cortex, epidermis and possible root cap or periderm preserved. (h) Detail of the vascular strand and endodermis showing the presence of Casparian strips (arrowhead). (i) Detail of the outer cortex and epidermis, epidermis highlighted by arrowhead. Bars: (a) 250 μm; (b) 250 μm; (c) 250 μm; (d) 100 μm; (e) 200 μm; (f) 200 μm; (g) 100 μm; (h) 25 μm; (i) 200 μm. Specimen accession codes: (a–d, g, h) NMS G.2025.5.7; (e, f) NMS G.2025.5.9; (i) NMS G.2025.5.6.

On the surface of the specimen is a large excavated area (Fig. 5a–c, red dashed line). The full extent and shape cannot be determined since it is excavated in the outer portion and therefore likely extended beyond the preserved specimen. The margins of this gallery have notched borders (Fig. 5d, arrowheads), and the excavated areas are filled with frass consisting of large (*c*. 500 μm) irregular pieces of secondary xylem randomly distributed in the galleries (Fig. 5). A few oval plugs of compacted frass, 1200–2000 μm wide, can be observed inside the large boring (Fig. 5a–c,e, green arrowheads). In bigger frass fragments, it is possible to identify the presence of white rot (Fig. 6f, arrowhead).

Alongside the larger galleries are small galleries, 100–400 μm wide, with irregular shape and borders (Fig. 5a,b yellow circles). These galleries are also excavated in white‐rotted wood (Figs 3b,c, 6a–c). In some areas of the wood, the small galleries appear broken and coalescing with the big gallery (Fig. 6c). The margins of these small galleries are smooth, in contrast to the large galleries (Fig. 6a–d). No frass remains were observed in these smaller galleries (Fig. 6a–e). Well‐preserved coprolites are abundant in the big galleries within the frass fragments (Figs 5d,e, 6f) and within smaller galleries. In the small galleries, coprolites are found either loose (Fig. 6a,b) or tightly packed (Fig. 6d,e). Coprolites are oval‐shaped, with smooth margins, and can be grouped into two size classes. Small coprolites (Figs 5d, 6a,b,d) are 43 (29–53) μm in diameter and 62 (48–74) μm long, with an aspect ratio of 1.43. Large coprolites (Fig. 6e,f) are 55 (48–63) μm in diameter and 90 (76–103) μm long, with an aspect ratio of 1.63.

Based on this evidence, we propose that the specimen was bored by two types of arthropods. We identified no evidence of reaction tissue surrounding the galleries and thus interpret that the fungal decay and arthropod borings occurred after the death of the branch or trunk (Harper *et al*., 2012; McLoughlin & Bomfleur, 2016; Sagasti *et al*., 2019, 2025; Li *et al*., 2024). The big galleries are consistent with the activity of wood‐boring beetles from the family Cerambycidae. Many cerambycid species are saproxylophagous and lay eggs on logs and fallen wood. The larvae then bore galleries from the bark to the heartwood and produce a heterogeneous frass composed of cylindrical pellets (*c*. < 1 mm long and 0.4 mm wide) mixed with small wood chips (Toriti *et al*., 2021). This damage pattern is known in both modern (Toriti *et al*., 2021) and fossil settings (Genise & Hazeldine, 1995; Sagasti *et al*., 2019; McLoughlin & Mays, 2022) and is similar to what we observe here.

An alternative arthropod that in modern settings produces damage consistent with our observations is the wood weevils of the Curculionidae family. In modern settings, they produce irregular to round galleries through the sapwood and the heartwood, filled with heterogeneous frass of wood chips and small fusiform pellets no longer than 0.2 mm (Toriti *et al*., 2021). The galleries of weevils can coalesce into one another as the activity of the colony progresses, similar to that observed in Fig. 6(c). Additionally, many curculionids are obligate mutualists with wood‐decaying ascomycetes and actively introduce mycelia and spores that aid in the digestion of secondary xylem (Farrell *et al*., 2001). The fungi can then be found inside galleries in close association with coprolites and frass, similar to what we observe here in Fig. 4(g). However, it seems unlikely that wood‐boring weevils caused this damage, as the origin of the weevil group is estimated to date from 20 Myr after that of the nurse log in the Barremian, Early Cretaceous (*c*. 130 Ma; Jordal *et al*., 2011).

The size, shape and location of the coprolites within the small galleries are consistent with the activity of oribatid mites (e.g. Labandeira *et al*., 1997; Kellogg & Taylor, 2004; Falcon‐Lang *et al*., 2015; McLoughlin & Bomfleur, 2016). Oribatid mites usually feed on dead and partially rotted plant material, including wood (Feng *et al*., 2010). Additionally, it is quite common to find an increase in coprolite size that reflects the ontogenetic sequence of two or more stages of metamorphosis in a single wood fragment (Labandeira *et al*., 1997). From this evidence, we identify the activity of two arthropods in this nurse log: the big galleries filled mostly with frass were likely the result of cerambycid beetles; and the small, coprolite‐filled galleries were likely bored by oribatid mites.

### The decayed wood provided a substrate for plant roots

Alongside the frass and well‐preserved coprolites within cavities in the secondary xylem are numerous roots (Fig. 5a–c, yellow arrows; Fig. 6g,h). The roots are broadly aligned parallel to the main woody axis and extend throughout the length of the specimen. Roots are 600–700 μm in diameter, circular in transverse sections, with preservation of some but not all tissue types. The stele is diarch with two poles of xylem (Fig. 6g–i), but there is no preserved phloem or evidence of secondary growth. Surrounding the stele is an endodermis, with rectangular cells in transverse section (127 × 172 μm) and Casparian strips on the radial walls (Fig. 6h arrowhead). The cortex is mostly decayed, with some circular parenchyma cells preserved (Fig. 6g,i). The epidermis is composed of a layer of rectangular to circular parenchymatic cells (Fig. 6i, arrowheads). In some cases, a multi‐layered tissue is preserved surrounding the root. This tissue is composed of compressed cells, arranged in concentric layers, and may correspond to the root cap or the periderm (Fig. 6g). We found no evidence of root hairs. The limited anatomical preservation makes taxonomic assignment of the roots challenging. Diarch roots of very similar form are known from a Triassic nurse log and were assigned as likely conifer roots and interpreted as an example of conifer wood acting as substrate for conifer roots (Daugherty, 1963). Similar examples where wood and roots are from the same group have also been described from other nurse logs (Césari *et al*., 2010, 2012; Decombeix *et al*., 2020; Vera & Perez Loinaze, 2022; Wang *et al*., 2024). However, due to the lack of anatomical features of taxonomic value preserved here and the conservative nature of root anatomy between taxa (Decombeix *et al*., 2020) we cannot assign the roots found in the Helmsdale nurse log. Regardless of the taxonomic affinity of the roots, their presence within the cavities, growing through frass and coprolites but without any evidence of arthropod damage, suggests they were established in the decayed log after the fungal and arthropod activity was well developed. Furthermore, they provide convincing evidence that the specimen acted as a substrate for the growth of other plants.

### A timeline for decay and multitrophic interactions in the Jurassic of Scotland

Helmsdale 1 represents an example of complex trophic associations in which a host woody plant acted as substrate for fungi, arthropods and other plants. Interactions like this demonstrate how individual samples can allow the reconstruction of complex food webs and biotic interactions in forest ecosystems from the past (Rößler, 2000; Slater *et al*., 2012, 2015; Strullu‐Derrien *et al*., 2012; Falcon‐Lang *et al*., 2015; McLoughlin & Bomfleur, 2016; McLoughlin & Strullu‐Derrien, 2016; Sagasti *et al*., 2019, 2025). The order of events is convincingly shown through the physical relationships preserved in the fossil. First, all decay processes appear to have occurred after the death of the branch or trunk as there is no evidence of any response or reaction tissue to all events such as traumatic resin ducts, local increases in cell wall thickness and the production of tyloses (e.g. Hudgins *et al*., 2004; Franceschi *et al*., 2005; Sagasti *et al*., 2019, 2025). Evidence of white pocket rot appeared to be the first main stage of decay. In modern systems, this is also known to occur early. White rots are very frequent in fallen logs of modern temperate forests. Some species of white rot fungi (e.g. *Phellinus pini*) are particularly efficient in degrading conifer woods after the log dies (Blanchette, 1991). The next process is evidence of arthropod borings. We propose that this occurred after the action of the white rot as frass filling the galleries includes xylem with indicative signs of white rot decay. In modern systems, the white rot fungi attack all cell wall components, including lignin, making the woody tissue more palatable for animals. Xylophagous arthropods, especially beetles, show a preference for boring galleries in fungal‐decayed woods (Owen *et al*., 2005; Bonello *et al*., 2006; Yee *et al*., 2006). The large galleries may well have been bored by similar beetles. The smaller galleries described in this study share characteristics in their shape, size and coprolite content with those excavated by oribatid mites. The presence of two size classes for the coprolites suggests the presence of two instars of these mites, although it is not possible to define whether they were inhabiting the wood simultaneously or whether it was the same group of mites that grew while feeding from this log. Finally, the bored wood showed evidence of growth of plant roots. Plant roots do not appear to be actively breaking through the wood but instead are making use of the arthropod borings. Decaying logs provide microsites favourable for the development of seedlings, especially in small‐seeded conifer species (Bače *et al*., 2012). The presence of litter and humus in the crevices of decaying logs provides microsites protected from desiccation and soil pathogens, as well as root competition (Lonsdale *et al*., 2008; Bače *et al*., 2012). Additionally, nitrogen content increases with decay, providing easily accessible nutrients to the establishing seedling (Zielonka, 2006). The presence of roots in white rotted Jurassic woods from Helmsdale is consistent with the findings of modern forest researchers and provides evidence of plant–plant interactions facilitated by fungi in the Jurassic.

### Conifer evolution and the rise of the nurse log strategy

There is extensive evidence from studies of living species that the nurse log strategy is widespread today in living natural and managed conifer forests (e.g. Harmon *et al*., 1986; Narukawa *et al*., 2003; Narukawa & Yamamoto, 2003; O'Hanlon‐Manners & Kotanen, 2004; Bače *et al*., 2012; Fukasawa, 2012, 2016). From these studies, it is clear that the prevalence of nurse logs is very important, contributing up to 95% of seedling establishment (McKee *et al*., 1982). The relevance of this ecological strategy can be illustrated in sub‐alpine forests where decayed wood debris only occupied 4% of the forest floor, yet 43% of seedlings from *Picea abies* were established on nurse logs (Zielonka, 2006). Studies like these have also demonstrated that for wood to act as a nurse log, it is likely that a level of initial decay is needed. Multiple studies have shown that the state of decay correlated with the number of established seedlings. This broadly followed a normal distribution: limited seedlings attached to fresh wood, or very late stages in decay; the maximum number of seedlings towards a mid‐state of decay (e.g. McKee *et al*., 1982; Narukawa *et al*., 2003; Fukasawa, 2012). This demonstrates the close relationship between the organisms that cause decay and germination. Studies focused on fungal rot have identified that some conifer species (i.e. *P. abies*, *Pinus densiflora*) grow preferentially on white rotted logs rather than on brown rotted ones (Fukasawa, 2015, 2016). This may be due to brown rot resulting in a lower pH microenvironment that interferes with the development of seedlings (Bače *et al*., 2012; Fukasawa, 2012, 2016). Each of these suggests there may have been a complex co‐evolution of the nurse log strategy with plants, fungi and animals. The long fossil record of conifers and the new fossil data here provide the opportunity to investigate the evolution of interactions.

We carried out a literature review of studies on fossil nurse logs spanning *c*. 300 Myr from the Carboniferous period to the Eocene (Table 2). Based on this, we found that of the nine studies reporting nurse logs, six are of conifer wood, and four had evidence of likely conifer roots growing in them. Of the nine examples of fossil nurse logs, seven have been described as roots growing in decayed woods, and one mentioned a decayed pith. However, only two studies describe with precision which type of fungal rot was present, whilst others describe the process in general terms, such as ‘spongy’ or ‘decayed’. Studies on fossil nurse logs have proposed a correlation between the nurse log strategy and waterlogged environments (e.g. Feng *et al*., 2022). Waterlogged soils have been suggested as triggering environmental stress that leads to the proliferation of the nurse log strategy (e.g. Molano *et al*., 2025). However, studies in modern conifer forests have demonstrated that the decay type is the single most significant factor affecting seedling establishment in conifer forests from different latitudes and climates and that white rot has a positive influence on the establishment of conifer seedlings (Fukasawa, 2016). The finding that fossil nurse logs are identified in wet environments may therefore represent a preservation bias towards fossilisation in these settings (Greb & DiMichele, 2006; Channing & Edwards, 2013), with decay of the wood still likely the most important factor in the prevalence of nurse logs.

**Table 2:** Literature review of fossil nurse logs, with this study highlighted in bold.

Age	Wood	Roots	Fungi (rot if known)	Arthropods	References
Upper Carboniferous	Cordaitalean	Cordaitalean	Yes	No	Wang *et al*. (2024)
Late Pennsylvanian – > 100 specimens, ‘several’ nurse logs	Cordaitalean	Cordaitalean	Yes	Yes, galleries and coprolites of oribatid mites	Césari *et al*. (2010, 2012)
Permian – 123 conifer stems analysed, 7 nurse logs	Conifer	Conifer and sphenophyllalean roots	Yes	Yes, corprolites of oribatid mites and probably millipedes	Feng *et al*. (2022)
Permian – 1 nurse log	Seed fern (Glossopteris)	Glossopterid	White pocket rot	Gallery of unclear origin is present (arthropods, fungi or previous roots are proposed)	Decombeix *et al*. (2020)
Triassic	Conifer	Likely conifer	‘Spongy’^a^	No	Daugherty (1963)
Jurassic	Conifer	Possible conifer	No	No	Molano *et al*. (2025)
**Jurassic – 20 specimens, 1 nurse log**	**Conifer**	**Diarch roots**	**Yes, white pocket rot**	**Yes. Possible beetle boring + galleries of oribatid mites**	**This study**
Cretaceous	Conifer	Conifer	Yes	No	Vera & Perez Loinaze (2022)
Eocene	Conifer	Conifer	Decayed pith + Ectomycorrhiza	No	Fairon‐Demaret *et al*. (2003)

^a^ A spongy texture is mentioned, but no explicit description of fungal rot is made.

The activity of wood‐boring arthropods, including oribatid mites, insects and millipedes, has also been proposed as a key element in the development of nurse logs, by generating conduits for root growth and releasing nutrients from the wood tissues (Feng *et al*., 2022). Our literature survey shows that in four of the nine examples, there is evidence of galleries in the wood. In all the cases where arthropod borings were present, there was also fungal decay affecting the plant tissues. This is consistent with modern forests in which fungal decay facilitates the activity of arthropods (e.g. Furniss & Carolin, 1977; Grimaldi & Engel, 2005; Owen *et al*., 2005; Toriti *et al*., 2021), and further supports our interpretation of the decay sequence for this Jurassic nurse log.

These findings indicate that the association between fungal decay, arthropod borings and the nurse log strategy has existed over a long period of time within conifer wood. The decay of the wood appears to play a key role in this ecological strategy, and a combination with the presence of open areas resulting from rotting or excavation of arthropods results in a favourable environment for the establishment and growth of seedlings. These results are consistent with the trends observed in modern conifer forests, where seedling establishment is more frequent in mid‐decayed logs with evidence of fungal rot and arthropod borings, but where the mechanical structure of the wood is still present (e.g. Bače *et al*., 2012; Fukasawa, 2012). It is interesting to note that the anatomical features of the secondary xylem of all fossil nurse logs, comprising cordaitalean, glossoperids and conifers are very similar. Additionally, all produce small seeds (Taylor & Taylor, 1987; Klavins *et al*., 2001; Marques de Souza & Iannuzzi, 2007). The prevalence of nurse logs within these groups of gymnosperms with similar wood and seeds suggests that the modern nurse log strategy seen most commonly in conifers may have evolved early and been widespread in their Palaeozoic relatives.

## Competing interests

None declared.

## Author contributions

AJH and AJS conceived the study, designed experiments, collected and generated new fossil preparations. AJS and SB acquired and processed data. JW and SB constructed the 3D model. AJH and AJS wrote the manuscript with inputs from SB and JW.

## Disclaimer

The New Phytologist Foundation remains neutral with regard to jurisdictional claims in maps and in any institutional affiliations.

## Data Availability

Data for this work can be found in the manuscript. The photogrammetry model is deposited on the Edinburgh University DataShare (doi: https://datashare.ed.ac.uk/handle/10283/8998). Fossil specimens described in this study are deposited in the collections of the National Museums Scotland, UK, with accession nos. NMS G.2025.5.1–5.9, NMS G.2025.6.1–5, NMS G.2025.7.1–7.5 and NMS G.2025.8.1.
